# Sub-acute Painless Vision Loss Secondary to Neuro-Ocular Syphilis

**DOI:** 10.7759/cureus.34044

**Published:** 2023-01-21

**Authors:** Amarjyo Randhawa, Kiratjyot Randhawa, Akshat Sood, Joseph Thirumalareddy, Sunil Nair

**Affiliations:** 1 Internal Medicine, CHI (Catholic Health Initiatives) Health Creighton University Medical Center - Bergan Mercy, Omaha, USA; 2 Medicine, Government Medical College, Amritsar, IND; 3 Internal Medicine, CHI (Catholic Health Initiatives) Creighton University, Omaha, USA; 4 Hospital Medicine, Internal Medicine, CHI (Catholic Health Initiatives) St. Alexius Hospital, Omaha, USA

**Keywords:** penicillin allergy, treponema pallidum, ocular syphilis, vision loss, syphilis

## Abstract

There has been a consistent increase in syphilis cases in the United States over the past two decades. The prevalence of neuro-ocular involvement in syphilis is rare; however, with the increase in the prevalence of syphilis in the United States, more frequent encounters with complications of syphilis may be expected. Here, we present a case of sub-acute painless vision loss secondary to neuro-ocular syphilis.

## Introduction

Ocular syphilis is a clinical manifestation of syphilis that can involve any structure of the eye [1]. Posterior uveitis and pan-uveitis are the most common presentations [1]. Other presentations include anterior uveitis, optic neuropathy, interstitial keratitis, retinitis, and retinal vasculitis [1]. Ocular syphilis can be associated with neurosyphilis. It can occur at any stage of syphilis, including primary and secondary [1]. Prompt identification of ocular syphilis is essential, as a delay in treatment can result in decreased visual acuity and permanent vision loss [1]. The incidence of syphilis has been increasing in the United States since the year 2000. In 2020, 13945 cases of all stages of syphilis were reported, including 41,655 cases of primary and secondary syphilis [2,3]. Quantifying the incidence and prevalence of neuro-ocular syphilis is challenging. Although syphilis is nationally notifiable, reporting of ocular syphilis was not required until the Centers for Disease Control and Prevention (CDC) introduced it on December 1, 2014 [1]. Syphilis surveillance and case investigation data for the years 2014-2015 were reviewed from eight jurisdictions in the United States of America. The results published in Morbidity and Mortality Weekly Report revealed that 0.6% of syphilis cases were consistent with ocular syphilis [4]. In a recent interview-based investigation of early syphilis (ES) cases, the self-reported prevalence of neurological and/or ≥1 ocular symptom was found to be 151 among 9123 cases (1.7%; 95% CI, 1.4%-1.9%). Among symptomatic ES cases, 36% (n = 54) reported neurologic symptom(s) only, 34% (n = 51) reported ocular symptom(s) only, and 30% (n = 46) reported having both neurologic and ocular symptoms. Among the 151 symptomatic cases, 22% reported vision changes(n=33), 30% reported blurry vision(n=46), 15% reported floaters (n=22), and 9% reported vision loss (n=14). Both studies report an increased predominance in the MSM (men who have sex with men) and HIV-positive populations [4,5].

It remains unknown whether the likelihood of ocular infection is related to certain strains of Treponema (T.) pallidum. A small study utilizing molecular typing of T. pallidum from patients with and without ocular syphilis found no clear evidence of a predominant oculotropic strain [6].

## Case presentation

**Figure 1:**
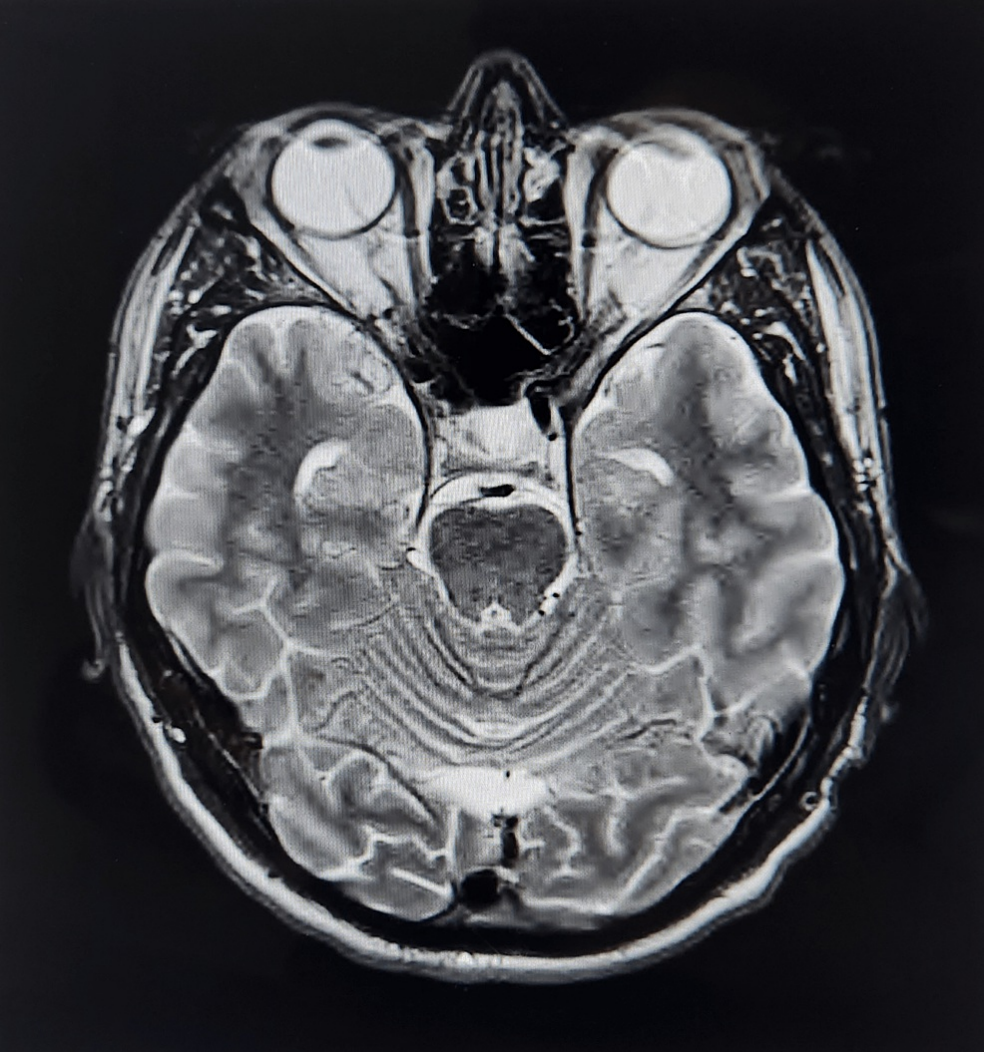
MRI brain

A 40-year-old Caucasian male with a past medical history of intravenous substance abuse (methamphetamine, cocaine), marijuana use, tobacco dependence, and historical chlamydia infection presented with sub-acute progressive vision loss in the left eye. The patient noticed blurry vision and the “wavy line sensation”; two weeks ago, it progressed to “blotchiness” and subsequently near-complete vision loss in the left eye. He denied any symptoms in his right eye. Associated symptoms include two episodes of subjective fever 10 days earlier followed by resolution, left ocular discomfort, headache, photophobia, neck pain, and imbalance, which he described as “walking like a drunk man.” The patient reported that he used to donate blood regularly until two years ago when he was informed that he had tested positive for syphilis. He did not seek or receive treatment for it. A review of systems was negative for ocular pain, eye discharge, eye redness, nausea, dizziness, syncope, focal weakness, numbness, tingling, chest pain, palpitations, genital ulcers, and skin rash. He reported a preference for female partners only. On physical exam, left eye vision was decreased (abnormal CN II exam). The patient was able to count and detect light. Photophobia and Kernig sign was present. CN III-XII abnormalities and focal neurological deficits were absent. Laboratory workup was remarkable for mild elevation in erythrocyte sedimentation rate (ESR) and positive qualitative syphilis screen. No significant abnormalities were noted on complete blood count (CBC), comprehensive metabolic panel (CMP), and C-reactive protein (CRP). There was a negative screen for hepatitis A, B, C, and HIV. A normal MRI brain excluded intracranial pathology (Figure 1).

Ophthalmology was consulted for urgent evaluation. A detailed ocular evaluation showed that left eye vision was limited to counting fingers at 3.5 ft. The right eye exam was normal with 20/20 vision. Pupils were 3.5 mm bilaterally with 1+ reaction to light. No afferent pupillary defect could be discerned with certainty, though there was a suggestion on the left. Visual field decreased in the left eye, normal in the right. Tonometry was unremarkable. Anterior segment exam revealed a subtle hint of limbal injection. No anterior segment inflammation or keratic precipitates were seen. Lenses, irises, anterior chamber conjunctiva, and cornea and lid margins were normal. On ophthalmoscopy, there was a blurring of optic disc margins and there was no optic cup. A similar blurring of optic disc margins was seen on the right eye as well, although not nearly as significant. Both discs were hyperemic. There was no evidence of retinal vasculitis or retinitis.

Treatment with IV penicillin G 24 million units/day pending results of confirmatory fluorescent treponemal antibody absorption (FTA-ABS), rapid plasma reagin (RPR) titer, and lumbar puncture results. The patient reported a historical allergy to penicillin in childhood, however, after a thorough, detailed history-taking from the patient’s mother, we ruled out anaphylaxis or significant allergic reaction to penicillin therefore desensitization was not required. Lumbar puncture showed a slight increase in WBC (CSF pleocytosis), RBC, mildly elevated protein, and normal glucose. A reactive FTA-ABS, serum RPR titer of 1:128, positive CSF VDRL (titer 1:2), and clinical presentation clinched the diagnosis of neuro-ocular syphilis with meningitis, optic neuropathy, and/or optic neuritis.

The patient reported improvement in vision within 48 hours of initiating treatment. A Jarish-Herxheimer reaction occurred within 24 hours, which was resolved with supportive measures. IV Penicillin G was continued for a total of 2 weeks. The patient endorsed remarkable improvement in vision during the primary care plan (PCP) clinic follow-up about two weeks after the completion of treatment. However, he expressed some residual photosensitivity, bright spots in his left eye, and double vision. Unfortunately, the patient did not show up for follow-up appointments at the ophthalmology and infectious disease clinics.

## Discussion

Per CDC guidelines, ocular syphilis represents clinical symptoms or signs consistent with ocular disease (i.e., uveitis, panuveitis, diminished visual acuity, blindness, optic neuropathy, interstitial keratitis, anterior uveitis, and retinal vasculitis) with syphilis of any stage [1]. Ocular syphilis manifestations (e.g., neuroretinitis and optic neuritis) can be isolated abnormalities or associated with neurosyphilis. All patients with involvement of the eye and reactive syphilis serology should undergo a full ocular exam including cranial nerve testing [1]. If clinical evidence of neurological involvement is noticed, a lumbar puncture with CSF evaluation is warranted prior to treatment [1]. Among patients with isolated ocular involvement (without cranial nerve or neurological abnormality), reactive syphilis, and confirmed ocular abnormalities on examination, CSF evaluation prior to treatment is not necessary. If ocular syphilis is suspected, an ophthalmologist should be consulted immediately for evaluation and a collaborative management plan [7]. Cases of ocular syphilis should be reported to your state or local health department within 24 hours of diagnosis [1]. Pre-antibiotic clinical samples (whole blood in ethylenediaminetetraacetic acid (EDTA) tubes, primary lesions, moist secondary lesions, CSF, or ocular fluid) should be saved and stored at -80°C immediately upon collection for molecular typing [1].

Ocular syphilis should be treated like neurosyphilis irrespective of the CSF study results. Recommended regimen constitutes aqueous crystalline penicillin G 18-24 million units per day administered as 3-4 million units IV every 4 hrs or continuous infusion for 10-14 days. If there is no concern of compliance, we can also consider procaine penicillin 2.4 million units IM once daily plus probenecid 500 mg QID, for 10-14 days. In patients with penicillin allergy, there are some data that support the use of ceftriaxone 1-2 g daily either IM or IV for 10-14 days as an alternative treatment for persons with neurosyphilis. If ceftriaxone cannot be used, a skin test or a specialist consult for penicillin desensitization may be necessary. Other regimens have not been adequately studied for the treatment of neurosyphilis. We do not have to repeat the lumbar puncture for a CSF study in immunocompetent patients or HIV patients who are on effective antiretroviral therapy (ART) treatment [7].

## Conclusions

This case highlights an atypical presentation with painless sub-acute vision loss consistent with clinical signs of CN II impairment, subtle signs of meningeal irritation, and absence of previously symptomatic syphilis. Although neuro-ocular manifestations of syphilis are rare, with the increase in the prevalence of syphilis in the United States, more frequent encounters with the complications of syphilis may be expected. It is critical for clinicians to be familiar with the presentation of neuro-ocular syphilis, as early treatment may prevent permanent visual deficits.
